# Attachment anxiety and cyberbullying victimization in college students: the mediating role of social media self-disclosure and the moderating role of gender

**DOI:** 10.3389/fpsyg.2023.1274517

**Published:** 2023-11-14

**Authors:** Xixi Yang, Yitong Huang, Benqian Li

**Affiliations:** School of Media and Communication, Shanghai Jiao Tong University, Shanghai, China

**Keywords:** attachment anxiety, cyberbullying, social media self-disclosure, cyberbullying victimization, Chinese college students

## Abstract

**Backgrounds and purpose:**

Cyberbullying is a globally prevalent social problem that threatens the wellbeing of young people. Despite a rising call for more research focused on cyberbullying victims, our understanding of the psychological and behavioral risk factors associated with cyberbullying victimization (CV) remains limited, especially among the Chinese population. However, such information is crucial for identifying potential victims and planning targeted educational and protective interventions. In this paper, we report an empirical investigation into how attachment anxiety (AA), social media self-disclosure (SMSD), and gender interplay with each other to influence CV.

**Methods:**

Cross-sectional survey data from 845 Chinese college students (Female = 635, *M_age_* = 18.7) were analyzed in SPSS PROCESS using Haye’s macro with the bootstrap method.

**Results:**

Our data support a moderated mediation model. First, SMSD partially mediates the positive relationship between AA and CV, which suggests individuals with high AA tend to engage in risky and excessive self-disclosure behavior on social media, which, in turn, expose them to an increased risk of cyberbullying. Second, gender moderates the direct AA-CV path and the second stage of the mediation path, making the effect of AA on CV appear more direct in males (i.e., not mediated by SMSD) and more indirect (i.e., fully mediated through SMSD) in females.

**Conclusion:**

The results contribute to an ongoing endeavor to better understand the psychological and behavioral mechanisms underlying CV and develop effective strategies to identify and protect vulnerable individuals.

## 1. Introduction

The development of information and communication technologies (ICTs) has enabled individuals to express themselves and engage in social activities on a diverse range of online platforms, such as Facebook, Twitter, Sina Weibo and TikTok. This, however, has also increased the risk of cyberbullying, which refers to repeated harm, humiliation, and harassment of other individuals or groups through online technology, in forms of name-calling, sending embarrassing pictures, sharing personal information or secrets without permission, spreading rumors, trickery, exclusion, and impersonation (Willard, 2007).

As a variation of traditional bullying that shares its many characteristics, cyberbullying has become a subject of growing social concern due to three distinct features. Firstly, cyberbullying could have more long-lasting influences than traditional bullying, as electronically communicated information is permanent and public, and can spread widely if not removed promptly (Vandebosch and Cleemput, 2008). Secondly, cyberbullying can be more pervasive and distressful than traditional bullying because ICTs enable continuous communication 24 h a day. In other words, it can be harder for victims to find relief (John et al., 2018). Indeed, cyberbullying victims exhibit 2.35 times the risk of self-harm, 2.15 times the risk of suicidal thoughts, and 2.10 times the risk of suicidal behavior compared with non-victims (John et al., 2018). Thirdly, the nature of computer-mediated communication on the Internet poses challenges for intervention. For instance, due to the anonymity and deindividuation effect, people are less likely to be self-disciplined and altruistic in the virtual than in the real world, which makes it difficult for cyberbullying interventions to identify, reach and educate perpetrators or bystanders in the virtual world (Slonje and Smith, 2008; Cassidy W. et al., 2013).

Given the above, there has been a call for a shift of research focus from perpetrators towards victims of cyberbullying to inform the development of interventions to mitigate their risks and suffering from cyberbullying. Part of the research along this line has been focused on identifying risk factors or predictors of cyberbullying victimization (CV). For instance, research has found that poor social skills and fear of rejection in interpersonal communication make some more vulnerable to cyberattacks than others (Kokkinos and Antoniadou, 2019). In addition, users’ self-disclosure behaviors, such as posting sensitive information and indiscreet statements, also increase their likelihood of becoming victims of cyberbullying (Chang et al., 2014). These studies show that cyberbullying most likely results from a combination of psychological and behavioral risk factors, the understanding of which is crucial to identifying vulnerable individuals and planning targeted interventions proactively.

Currently, cyberbullying research is primarily focused on children and adolescents and is mostly conducted in developed countries. Little attention has been paid to college students, many of whom are also suffering from cyberbullying (Francisco et al., 2015; Tsitsika et al., 2015). Even less attention has been dedicated to low- and middle-income countries, where there is an increased prevalence of cyberbullying among young people and thus an urgent need for cyber-bullying research to understand the risk factors (Zhu et al., 2021). In China, cyberbullying victimization is particularly prevalent among college students [e.g., 71.9% according to Guangzhou Youth Palace, 2016 and 72.9% according to Xiao and Wong, 2013]. This is concerning yet understandable, because college is an important educational cycle that coincides with the life phase of “emerging adulthood” characterized by the need for increased autonomy and responsibility, along with increased demands for academic training directly tied to career prospects (Martínez-Monteagudo et al., 2020). In China, during this life phase, a typical Chinese college student may leave a protective family for the first time while still developing skills required for coping with various challenges that come with the new environment, including but not limited to the separation from parents and old friends and the creation of new social circles. For many Chinese college students, this transitional phase into university life is further complicated by the significant socio-economic disparities between urban cities and rural areas, as well as between the eastern and western regions of China. In a way, ICTs and social networking sites (SNSs) could facilitate such transition by supporting college students’ self-expression and socialization. However, it comes with the risk of being exposed to potential bullies online. As such, it is imperative to delve into the issue of cyberbullying within the context of this rapidly evolving society, which would offer valuable insights that may not only inform development of locally relevant interventions but also contribute to a broader cross-cultural understanding of cyberbullying worldwide.

In view of the above, in this study, we focus on factors and mechanisms that contribute to cyberbullying victimization in Chinese college students. In particular, the objective of this study is to investigate how attachment anxiety, social media self-disclosure, and gender interplay with each other to influence cyberbullying victimization.

## 2. Literature review

### 2.1. Attachment anxiety and cyberbullying victimization

Attachment anxiety (AA) stems from the attachment theory, which defines attachment as the emotional bonds we form with significant others including parents, close friends, and partners. The theory posits that the earliest bonds one forms with their caregivers in infancy bear long-term influence on interpersonal relationships in adulthood (Cassidy J. et al., 2013). Individuals who experience insecure attachments in childhood are prone to high attachment anxiety in relationships later in life, characterized by a strong desire for closeness and fear of rejection (Smith et al., 1999), as well as a positive view of others and a negative view of the self (Gradinger et al., 2009). Moreover, people with high attachment anxiety are less capable of establishing and maintaining interpersonal relationships (Simpson and Rholes, 2017).

There is ample research suggesting the association between attachment anxiety and bullying victimization. A possible explanation is that bullies tend to attack those with high attachment anxiety and fear of negative evaluations, as they are more likely to be upset by aggressive behaviors, which allows bullies to achieve their bullying goals (Navarro et al., 2011). Moreover, bullying is more likely to occur when there is a power imbalance between two parties in an interpersonal relationship (Kokkinos, 2013). As individuals of anxious attachment style tend to have lower self-esteem, develop poorer social skills and display more fear of social rejections (Mikulincer and Shaver, 2016), they are less capable of maintaining a fair interpersonal relationship and hence more likely to become victims of bullying (Navarro et al., 2018). We believe similar mechanisms underlying victimization in traditional bullying should hold for cyberbullying. Therefore, we hypothesize that:

*H1*: Attachment anxiety is positively associated with cyberbullying victimization.

### 2.2. The role of social media self-disclosure

Social media self-disclosure (SMSD) refers to individuals using text, pictures, and videos to convey information about themselves on social media (Ledbetter et al., 2010). To some extent, social media self-disclosure does allow individuals with attachment anxiety and poor social skills to reach out to a larger group of audience without boundaries and satisfy social needs that are unmet in the physical world (Lai and Yang, 2014). First, social media provides an online mode of mediated interaction where individuals can post images, moods, and even their personal information at any time. Therefore, individuals with poor social skills can use social media as a platform to express themselves, make friends and alleviate feelings of loneliness (Luo and Hancock, 2020). Second, the anonymity of social media does allow individuals with attachment anxiety to connect with others while avoiding the potentially anxiety-provoking aspects of face-to-face interpersonal communication (Eichenberg et al., 2017). For individuals with attachment anxiety, their sensitivity and vulnerability in interpersonal interactions may hinder their ability to form and maintain relationships in the real world; as a result, they may turn to strangers online for compensatory emotional supports (Konok et al., 2016). In this regard, social media provides a more relaxing communication environment, in which they are more likely to engage in self-disclosure (Cheng et al., 2019). Indeed, prior research has suggested that people with attachment anxiety use social media more frequently and care more about how they are perceived by others online (Oldmeadow et al., 2013); they also have stronger desires for social interactions and disclose more personal information online (Aharony, 2016). Therefore, we hypothesize that:

*H2*: Attachment anxiety is positively associated with social media self-disclosure.

Much research on cyberbullying victimization has indicated that risky internet use (such as sharing personal information with strangers online) was a significant risk factor for cyberbullying victimization over time. Chatting with strangers and sharing personal information in the cyberspace may expose oneself to potential bullies, even if the content of disclosure seems neutral (Walrave et al., 2012). Because of the anonymity and deindividuation effect of the Internet, some may post indiscreet statements and extreme views on sensitive topics and post photos with themselves in revealing attire (Peluchette et al., 2015), a phenomenon known as the online disinhibition effect (Suler, 2004). However, disclosing such information can have negative consequences, including but not limited to reputation damage and increased risks of sexual harassment (Kowalski et al., 2014). Therefore, we hypothesize that:

*H3*: Social media self-disclosure is positively associated with cyberbullying victimization.

When it comes to potential mediating mechanisms between attachment anxiety and cyberbullying victimization, the compensatory media use theory can be drawn upon, which posits that individuals who have personality characteristics that lead to an impoverished social life are more likely to seek social gratifications from media use (Tsao, 1996; Zywica and Danowski, 2008). In line with this, scholars have referred to variables such as loneliness, social anxiety and need to belong collectively as social compensation variables and found such needs positively predict pursuit of social gratifications in media consumption (Rosaen and Dibble, 2016). We argue that the same mechanism would apply to the context of social media, in terms of motivating those high in attachment anxiety to seek social gratifications through excessive and even risky social media use, which would in turn increase their likelihood of becoming victims of cyberbullying. Therefore, we hypothesize that:

*H4*: Social media self-disclosure mediates the effect of attachment anxiety on cyberbullying victimization.

### 2.3. The moderating effect of gender

Gender is often considered an important moderator in studying power dynamics and attachment styles in interpersonal relationships. A recent study on cyberbullying showed that neuroticism positively predicted cyberbullying victimization and the association was stronger in males than females (Zhang et al., 2020). Neuroticism is a personality trait characterized by emotional instability, anxiety, fear, and sadness, which are also prominent features of attachment anxiety (Donges et al., 2015). Given the strong correlation between attachment anxiety and neuroticism, the association between attachment anxiety and cyberbullying victimization may vary by gender in the same direction as that between neuroticism and cyberbullying victimization. Therefore, we hypothesize that:

*H5*: Gender moderates the relationship between attachment anxiety and cyberbullying victimization in the way that the relationship is stronger in males than in females.

Attachment anxiety may have different effects on social media self-disclosure depending on gender. The extant literature also provides evidence for the potential gender difference in the relationship between attachment anxiety and self-disclosure on social media platforms. Research shows that females with high levels of attachment insecurity tend to disclose more information on Facebook than their male counterparts (Aharony, 2016). This phenomenon may be attributed to traditional gender role expectations, which encourage females to express themselves and seek social support more than males (Eagly, 2013). It is also observed that females utilize social media platforms for social and emotional purposes (Dhir et al., 2016), whereas males use social media primarily for informational purpose, such as reading and sharing political news (Chadwick and Vaccari, 2019). Therefore, it is reasonable to hypothesize that:

*H6*: Gender moderates the relationship between attachment anxiety and social media self-disclosure in the way that attachment anxiety has a stronger effect on social media self-disclosure in females than in males.

Gender difference also exists in how one responds to unfavorable situations such as cyberbullying after they disclose themselves. Generally speaking, when faced with bullying, females tend to conform to their feminine norm by concealing their uncomfortable feelings toward attacks and being less confrontational, whilst males may choose to fight back (Mahalik et al., 2005; Werner and Hill, 2010). As it is still unclear which coping style may aggravate the situation and increase the severity of cyberbullying, the direction of gender moderation in this relationship is unknown. Therefore, we hypothesize that:

*H7*: Gender moderates the relationship between social media self-disclosure and cyberbullying victimization.

Based on the above, a moderated mediation model with the following hypothesis was proposed:

*H8*: Gender moderates both the direct and indirect relationships between attachment anxiety and cyberbullying victimization.

## 3. Methods

### 3.1. Study design

We collected data for this cross-sectional study using a web-based survey with self-report psychological measures of attachment anxiety, social media self-disclosure, cyberbullying victimization and other relevant information (e.g., gender, age, discipline, educational level, and social media usage). The survey was created in wjx.cn, a well-known online survey website in China and distributed on social media platforms like Weibo and WeChat. An informed consent form was attached to the questionnaire as the introduction page to inform participants of the aim, procedure, risks and benefits of the study. Participants indicated their consent to participant in the survey by clicking the “agree” button to proceed to the survey. After completing the questionnaire, they were compensated with a WeChat Hongbao with a random amount between 1 and 10 RMB. The study design was approved by the Institutional Review Board of Shanghai Jiao Tong University.

### 3.2. Participants

We employed the snowballing sampling method. The survey was initially distributed via the wjx.cn subject pool as well as the researchers’ social media accounts. The first section of the survey consisted of 3 screening questions based on the inclusion criteria: “Have you used social media (including Sina Weibo, WeChat, QQ)?” “Are you currently a college student (including undergraduate, postgraduate, associate degree)?” and “Have you ever been the target cyberbullying?” Respondents would be deemed eligible and directed to the next section only if they reported “yes” to all three questions. 870 Chinese college students completed the survey, among which 15 were invalid because of not using social media. This resulted in a total of 845 valid respondents (210 male, 635 female, *M_age_* = 18.7). Table 1 presents a summary of participants characteristics.

**Table 1:** Participant characteristics (*N* = 845).

		*N (%)*
Gender	Male	210 (24.9)
	Female	635 (55.1)
Age	<18 years	23 (2.7)
	18–20 years	505 (59.8)
	21–23 years	176 (20.8)
	24–26 years	89 (10.5)
	>26 years	52 (6.2)
Discipline	Humanities and Arts	438 (51.8)
	Social Sciences	174 (20.6)
	Natural Sciences	32 (3.8)
	Engineering Sciences	158 (18.7)
	Medicine and Life Sciences	43 (5.1)
Educational level	Junior college students	420 (49.7)
	Undergraduate students	217 (25.7)
	Postgraduate students	150 (17.8)
	Doctoral students	58 (6.9)
Average monthly social media use	1–6 days	83 (9.8)
	7–12 days	39 (4.6)
	13–18 days	57 (6.7)
	19–24 days	99 (11.7)
	25–31 days	567 (67.1)
Average daily social media use	<1 h	66 (7.8)
	2–4 h	279 (33)
	5–7 h	253 (29.9)
	8–10 h	130 (15.4)
	>10 h	117 (13.8)

### 3.3. Measures

#### 3.3.1. Social media self-disclosure

The Self-Disclosure Scale revised by Lai and Yang (2014) was used to measure the extent to which Chinese college students disclosed personal information online (eg, “I often express my personal opinions on social media”). It contained 12 items to be rated on a 5-point Likert scale (1 = strongly disagree, 5 = strongly agree). The Cronbach’s α of this scale was 0.81.

#### 3.3.2. Cyberbullying victimization

The Cyberbullying Victimization Scale (Patchin and Hinduja, 2010) was used to measure the extent to which Chinese college students experience cyberbullying victimization. The adapted scale consisted of 9 items (eg, “Someone has sent me insulting messages or comments in the last month”) to be rated on a 5-point Likert scale (1 = never, 5 = most of the time). The Cronbach’s α of this scale was 0.95.

#### 3.3.3. Attachment anxiety

The “attachment anxiety toward friends” 3-item subscale (eg, “I’m afraid my friend may abandon me”) from the Chinese version of Experience in Close Relationships—Relationship Structures Scale (ECR-RS) revised by Li and Kato (2006) was used. The items were rated on a 5-point Likert scale (1 = strongly disagree, 5 = strongly agree) and showed good validity and reliability (Fraley et al., 2011). The Cronbach’s α of this scale was 0.78.

### 3.4. Statistical analyzes

After being collected, data were analyzed in SPSS version 22. The mediation model (Model 4) and the moderated mediation model (Model 59) from Hayes were tested using the PROCESS macro (Hayes, 2012). Bias-corrected 95% confidence intervals (BC 95% CIs) were obtained by drawing 5,000 samples to test direct and indirect effects. When a bootstrapped BC 95% CI does not include zero, it indicates the parameter is statistically significant and the effect exists.

## 4. Results

### 4.1. Descriptive statistics and bivariate correlations

Table 2 presents the descriptive statistics and Pearson’s correlation analysis results of all variables addressed in hypotheses (e.g., attachment anxiety, social media self-disclosure, and cyberbullying victimization). The bivariate correlations between the three variables were all statistically significant, lending support to potential mediation effect.

**Table 2:** Correlation analysis of main variables.

Variable	*M*	SD	1.	2.	3.
1. AA	3.08	0.92	1		
2. SMSD	2.76	0.56	0.093**	1	
3. CV	1.36	0.63	0.081*	0.140**	1

*p < 0.05, **p < 0.01, ***p < 0.001. AA, attachment anxiety; SMSD, social media self-disclosure; CV, cyberbullying victimization.

### 4.2. The mediating effect of social media self-disclosure

To examine H1, H2, H3, and H4, we tested model 4 from Hayes’s PROCESS macro, which was a mediation test based on Bootstrapping (Hayes, 2017). Attachment anxiety was entered as the predictor variable, cyberbullying victimization as the outcome variable, social media self-disclosure as the mediator, and gender, age, education level, average monthly social media use and average daily social media use as covariates in the model. As shown in the regression table (see Table 3), the total effects of attachment anxiety on cyberbullying victimization were significant (*β* = 0.07, *p* < 0.001, 95% CI = [0.03, 0.12]), indicating that respondents with higher attachment anxiety had been victimized more in cyberbullying, which supported H1. Attachment anxiety was significantly and positively associated with social media self-disclosure (*β* = 0.06, *p* < 0.001, 95% CI = [0.02, 0.10]). Social media self-disclosure was significantly and positively associated with cyberbullying victimization (*β* = 0.14, *p* < 0.001, 95% CI = [0.06, 0.21]), supporting H2 and H3. After including social media self-disclosure in the model, attachment anxiety still had a significant effect on cyberbullying victimization (*β*  = 0.06, *p*  < 0.001, 95% CI = [0.02, 0.11]), with reduced effect size, indicating partial mediation and lending support to H4. As age, education level, average monthly social media use, and average daily social media use showed no significant effect, they were not be included in subsequent analysis.

**Table 3:** Regression analysis of the mediating role of social media self-disclosure.

Regression equation		Fit indices			Significance of coefficients			
Outcome variables	Predictor variables	*R*	*R* ^2^	*F*	*β*	LLCI	ULCI	*t*
CV		0.264	0.070	10.473***				
	Gender				0.363	0.257	0.468	6.751***
	Age				−0.050	−0.118	0.018	−1.450
	Educational level				0.042	−0.028	0.113	1.179
	AMSMU				−0.026	−0.060	0.008	−1.526
	ADSMU				0.024	−0.014	0.062	1.241
	AA				0.071	0.026	0.116	3.079**
SMSD		0.217	0.047	6.906***				
	Gender				0.018	−0.076	0.112	0.373
	Age				−0.023	−0.084	0.038	−0.745
	Educational level				0.110	0.047	0.173	3.422**
	AMSMU				0.029	−0.001	0.059	1.898
	ADSMU				0.010	−0.024	0.044	0.583
	AA				0.056	0.016	0.097	2.738**
CV		0.289	0.083	10.864***				
	Gender				0.360	0.255	0.465	6.751***
	Age				−0.047	−0.115	0.020	−1.369
	Educational level				0.028	−0.043	0.098	0.766
	AMSMU				−0.030	−0.064	0.003	−1.763
	ADSMU				0.023	−0.015	0.060	1.178
	AA				0.063	0.018	0.108	2.755**
	SMSD				0.135	0.060	0.210	3.516***

AMSMU, average monthly social media use; ADSMU, average daily social media use. *p < 0.05, **p < 0.01, ***p < 0.001.

Table 4 presents the results of the bootstrap test. Attachment anxiety had both direct and indirect effects on cyberbullying victimization, with 11.27% of the total effect mediated through social media self-disclosure (*β* = 0.01, *p* < 0.001, 95% CI = [0.00, 0.02]), which confirmed H4.

**Table 4:** Total effect, direct effect and mediating effect.

	*β*	SE	LLCI	ULCI	Relative effect value
Total effect	0.072	0.023	0.026	0.116	
Direct effect	0.063	0.023	0.018	0.108	88.732%
Mediating Effect	0.008	0.004	0.001	0.017	11.268%

### 4.3. The role of gender

A series of analyzes were performed to test H5-7, which focused on the moderating role of gender.

#### 4.3.1. Gender differences in main variables

First, results of independent sample *t*-tests suggested female respondents had significantly higher attachment anxiety but significantly lower social media self-disclosure and cyberbullying victimization than their male counterparts (Table 5), suggesting the plausibility of a moderated mediation model.

**Table 5:** Independent sample *t*-test.

Variables	Total sample (*N* = 845)	Male (*N* = 210)	Female (*N* = 635)	*t-*value	*p-*value
	*M(SD)*	*M(SD)*	*M(SD)*		
1. AA	3.08(0.92)	2.95(0.94)	3.12(0.92)	−2.30	0.02
2. SMSD	2.76(0.56)	2.83(0.56)	2.73(0.55)	2.29	0.02
3. CV	1.36(0.63)	1.61(0.79)	1.27(0.54)	5.83	0.000

#### 4.3.2. Moderating effect of gender in bivariate relationships

**Figure 1:**
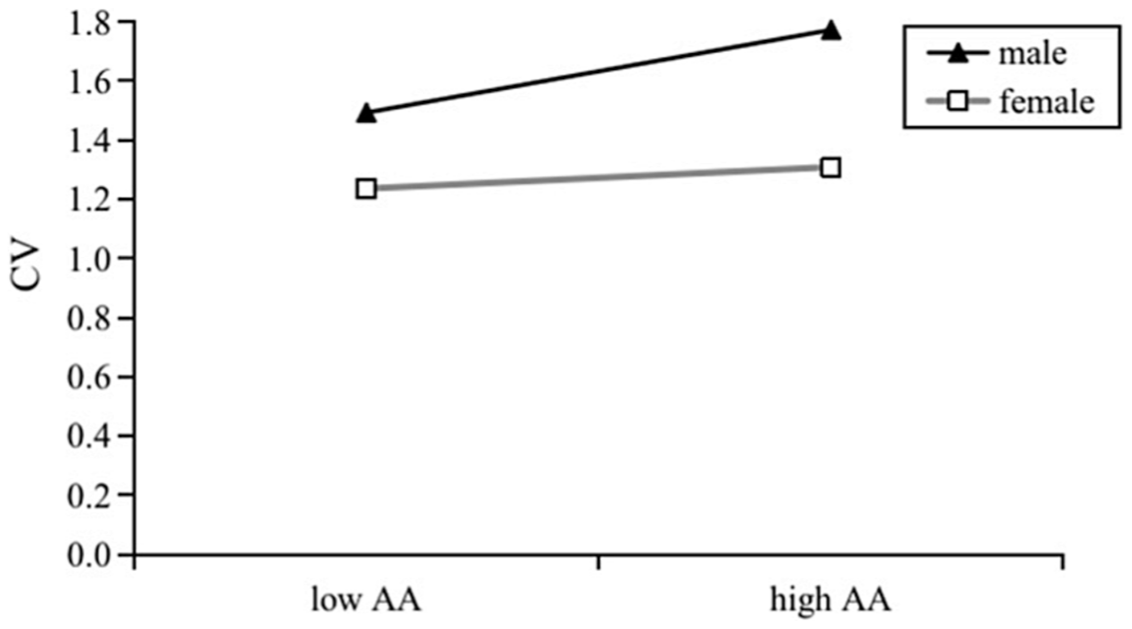
Effects of AA on CV by gender.

**Figure 2:**
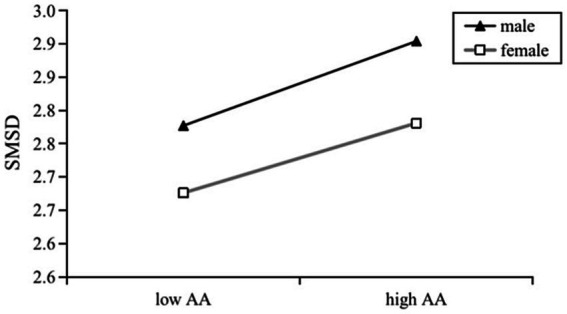
Effects of AA on SMSD by gender.

**Figure 3:**
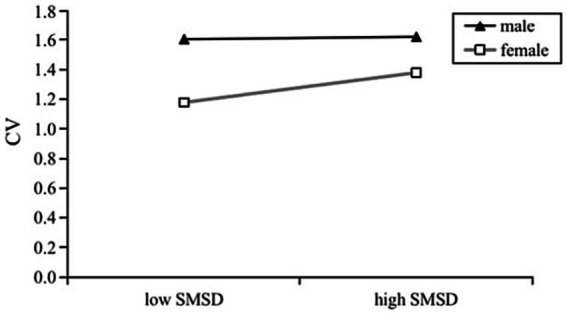
Effects of SMSD on CV by gender.

We then examined the moderating role of gender in pairwise linear relationships between the three main variables by executing model 1 from Hayes’s in PROCESS macro. Gender was dummy-coded (female = 0; male = 1). Results are presented in Figures 1–3.

As shown in Figure 1, there was significant interaction between attachment anxiety and gender in predicting cyberbullying victimization (*β* = 0.113, *p* = 0.031, 95% CI = [0.010, 0.215]), which meant gender moderated the AA-CV relationship. Moreover, post-hoc analysis found the simple slope was only significant in males (*β* = 0.152, *t* = 3.383, *p* < 0.001, 95% CI = [0.064, 0.240]) but not significant in females (*β* = 0.039, *t* = 1.493, *p* = 0.136, 95% CI = [−0.012, 0.091]). Therefore, H5 (attachment anxiety would have a stronger effect on cyberbullying victimization in males than in females) was supported.

In contrast, Figure 2 shows the AA-SMSD relationships did not differ significantly between gender, confirmed by the non-significant coefficient for the interaction term (*β* = 0.012, *p* = 0.806, 95% CI = [−0.081, 0.105]). Therefore, the hypothesis that attachment anxiety would have a stronger effect on social media self-disclosure in females than in males (H6) was rejected.

Although the interaction between social media self-disclosure and gender in predicting cyberbullying victimization was only marginally significant (*β* = −0.166, *p* = 0.057, 95% CI = [−0.336, 0.005]), post-hoc analysis found the simple slope was significant for females (*β* = 0.180, *t* = 4.141, *p* < 0.001, 95% CI = [0.095, 0.266]) but not significant for males (*β* = 0.015, *t* = 0.195, *p* = 0.846, 95% CI = [−0.133, 0.162]) (Figure 3). Based on the above, we could claim H7 was supported in the sense that significant gender difference exists in the SMSD-CV relationship.

#### 4.3.3. Moderated mediation

**Figure 4:**
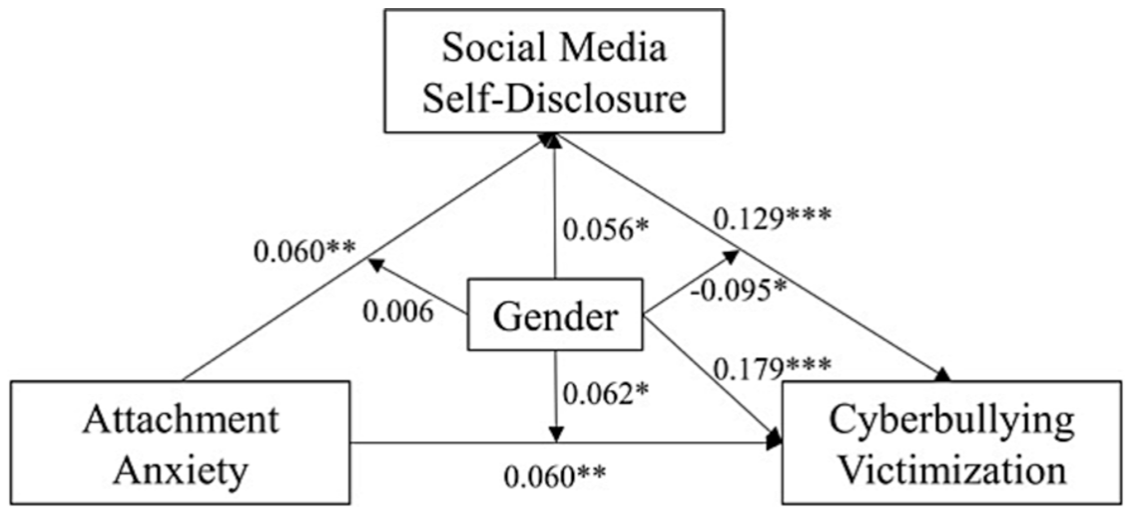
Results of the research model. *p < 0.05, **p < 0.01, ***p < 0.001.

H8 were tested by estimating a moderated mediation model (Model 59) in the PROCESS macro from Hayes. The resulting equation and model are presented below (see Table 6 and Figure 4). Gender was effect-coded (female = −1, male = 1), which meant the coefficients for attachment anxiety and social media self-disclosure represented the grand mean of their main effects in the whole sample.

**Table 6:** Moderated mediation model.

Regression equation		Fit indices			Significance of regression coefficient			
Outcome variables	Predictor variables	*R*	*R* ^2^	*F*	*β*	LLCI	ULCI	*t*
SMSD	AA	0.127	0.016	4.580**	0.060	0.019	0.100	2.896**
	Gender				0.056	0.013	0.099	2.529*
	AA*Gender				0.006	−0.041	0.052	0.246
CV	AA	0.296	0.088	16.082***	0.060	0.016	0.105	2.652**
	SMSD				0.129	0.055	0.202	3.412***
	Gender				0.179	0.131	0.226	7.322***
	AA*Gender				0.062	0.011	0.113	2.380*
	SMSD*Gender				−0.095	−0.181	−0.010	−2.195*

Gender is effect-coded, female = −1, male = 1. *p < 0.05, **p < 0.01, ***p < 0.001.

The results revealed gender moderation of both direct and indirect effects of attachment anxiety on cyberbullying victimization, supporting H8. To begin with, attachment anxiety had a weak but significant positive direct effect on cyberbullying victimization (*β* = 0.06, *t* = 2.65, *p* = 0.008, 95% CI = [0.02, 0.11]), which was moderated by gender (*β* = 0.06, *t* = 2.38, *p* = 0.02, 95% CI = [0.01, 0.11]). It was further revealed that gender moderated part of attachment anxiety’s mediated effect on cyberbullying victimization – the second stage of mediation (i.e., the SMSD-CV path) (*β* = −0.10, *t* = −2.20, *p* = 0.029, 95% CI = [−0.18, −0.01]), but not the first stage (i.e., the AA-SMSD path) (*β* = 0.01, *t* = 0.25, *p* = 0.806, 95% CI = [−0.04, 0.05]). In brief, our data supported a second-stage moderated mediation model.

**Figure 5:**
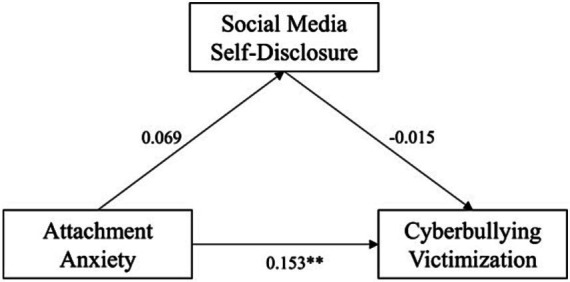
Pathway of attachment anxiety’s effect on cyberbullying victimization in males. *p < 0.05, **p < 0.01, ***p < 0.001.

**Figure 6:**
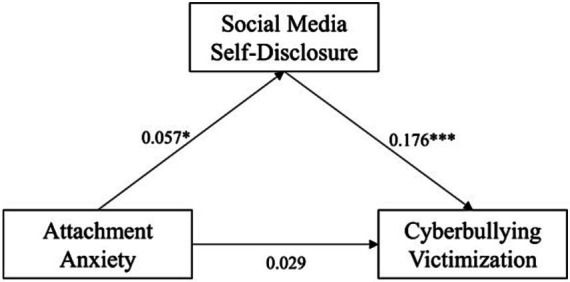
Pathway of attachment anxiety’s effect on cyberbullying victimization in females. *p < 0.05, **p < 0.01, ***p < 0.001.

Turning now to the resulting regression equation, we can see gender influenced cyberbullying victimization via several means. First, gender influenced cyberbullying victimization by moderating the direct, unmediated AA-CV path, which was amplified in males (β*_simple slope_* = 0.15, *t* = 3.41, *p* < 0.001) but non-significant in females (β*_simple slope_* = 0.03, *t* = 1.12, *p* = 0.264). Second, gender influenced cyberbullying victimization by moderating the SMSD-CV path, which was significant in females (β*_simple slope_* = 0.18, *t* = 4.04, *p* < 0.001) but non-significant in males (β*_simple slope_* = −0.02, *t* = −0.20, *p* = 0.84). The above suggested the pathway of attachment anxiety’s effect on cyberbullying victimization could be more direct in males and indirect in females, as confirmed by sub-group analyzes (see Figures 5, 6). Last but not least, gender, as a covariate, exerted a significant direct effect on cyberbullying victimization (*β* = 0.18, *t* = 7.32, *p* < 0.001, 95% CI = [0.13, 0.23]) unexplained by other variables we had included in the model, which meant males would have significantly higher mean cyberbullying victimization than females, after controlling for other variables.

## 5. Discussion

### 5.1. Principal findings

Unlike most existing cyberbullying research which focuses on perpetrators’ traits, in this study, we have specifically investigated victims and examined how a psychological construct stemming from the attachment theory (i.e., attachment anxiety) has influenced cyberbullying victimization via the mediation of an online behavior (i.e., social media self-disclosure). To our knowledge, this is the first published empirical study that has sought to uncover the mechanism underlying cyberbullying victimization through the lens of attachment theory in a Chinese sample. Previous research has looked into the relationships between attachment anxiety and cyberbullying victimization (Worsley et al., 2018b), attachment anxiety and social media self-disclosure (Chen et al., 2019), and social media self-disclosure and cyberbullying victimization (Choi and Lee, 2017) in separation, but none has considered them as a whole or paid sufficient attention to college students in low- to middle-income countries (Zhu et al., 2021). Our results corroborate previous research showing positive pairwise AA-CV (Varghese and Pistole, 2017), AA-SMSD (Morey et al., 2013), and SMSD-CV correlations (Dredge et al., 2014), and add to this existing body of literature with evidence on the mediating role played by social media self-disclosure between attachment anxiety and cyberbullying victimization. This reveals that individuals with high attachment anxiety tend to engage in risky and excessive self-disclosure behavior on social media, possibly in an attempt to seek out connections with others, which, however, exposes them to an increased risk of cyberbullying.

Regarding gender differences, some previous research shows males have significantly higher cyberbullying victimization than females (Akbulut and Eristi, 2011; Rao et al., 2019) while other research suggests the opposite (Alhajji et al., 2019; Aizenkot and Kashy-Rosenbaum, 2021). Our finding is in line with the former. But we have moved beyond that to explore reasons underlying this gender difference from the perspective of attachment theory by examining moderation in the various pathways in the mediation model. It is worth noting that although females show significantly higher attachment anxiety than males, it does not lead to higher cyberbullying victimization than males; moreover, the total effect of attachment anxiety on cyberbullying victimization is stronger in males than in females. Based on that, we suspect females may cope with attachment anxiety differently from males with some distinct mechanisms, which effectively buffer females’ risks for cyberbullying victimization. Next, we will elaborate on this by interpreting the moderated mediation model resulting from this study.

To begin with, contrary to our hypothesis, gender does not moderate the AA-SMSD relationship. One possible explanation could be the disappearance of gendered expectations on self-disclosure, as men are increasingly allowed and encouraged to disclose themselves nowadays (Sheldon, 2013). Some research has even found males are more likely to disclose themselves to recently added Facebook friends than females (Valkenburg et al., 2011). Indeed, as both males and females have a high need for connectedness and intimacy during college, online communication is seen as useful means for seeking out new connections, especially for those with high attachment anxiety and difficulty with social life in the physical world (Worsley et al., 2018a).

Nevertheless, what gender does moderate is the second stage of the mediation, namely the SMSD-CV path, which is significant in females but non-significant in males. This can be possibly explained by the gender difference in the contents of self-disclosure online. Previous research has found although social media self-disclosure exists in both genders, females tend to disclose more about themselves, and relationships with family and friends (Bond, 2009), personal views and feelings (Bane et al., 2010), and intimate topics (Valkenburg et al., 2011), because they believe this type of communication helps attract attention from others and build intimacy (Al-Saggaf and Nielsen, 2014). However, they may not realize that the sensitive content they share can make them targets of cyberbullying.

The second path via which gender exerts an influence on cyberbullying victimization is through moderating the direct AA-CV path. Attachment anxiety has a stronger impact on cyberbullying victimization in males than females. This suggests although the indiscreet social media self-disclosure behavior of many females may have put them at increased risk of cyberbullying victimization, there is some protective mechanism that buffers the AA-CV relationship for females despite their higher mean attachment anxiety than males. Perhaps females have applied some effective strategies to cope with negative emotions resulting from attachment anxiety (Razjouyan et al., 2018), including maintaining more intimate relationships and active communication with parents remotely (Larraaga et al., 2016), and initiating more help-seeking behaviors that lead to more social support (Heerde and Hemphill, 2018), all of which are associated with reduced risks for cyberbullying victimization. In this study, we fit our data to a moderated mediation model, but future work may potentially test a mediated moderation model, with a focus on identifying psychological, behavioral, and socio-environmental variables such as emotional intelligence (Martínez-Monteagudo et al., 2019), optimism (Chen et al., 2017), coping styles (Horner et al., 2015), help-seeking behaviors (Ševčíková et al., 2015), peer attachment (Wright et al., 2021), instructive parental mediation (Wright and Wachs, 2018) that could have potentially underlain the moderation effect of gender.

### 5.2. Limitations and implications for future work

Several limitations should be noted when interpreting our findings. Firstly, although the use of a cross-sectional survey serves to gauge the extent of three main variables of interest from a large sample simultaneously, the method is inherently associated with predictive limitations when it comes to causal inference (Chan et al., 2021). We have employed a theory-driven approach to generate and test hypotheses, which partially addresses this limitation. Case–control, cohort, and interventional studies may be conducted in the future to establish causal relationships between the variables.

Secondly, the current sample is unbalanced, as the proportion of females is three times that of males, which may limit the generalizability of the present findings to the general population. Although this is a common limitation in online survey studies due to gender differences in willingness to respond (Smith, 2008), we sought to tackle this limitation at least partly by including gender as a covariate to control for the potential effect of gender in the study.

Thirdly, the variance explained by our overall model is relatively small, suggesting the potential of other constructs outside of attachment theory that may explain cyberbullying victimization. As bullying is a complex psychosocial phenomenon, we did not expect attachment theory alone to have superb explanatory power. Further work is needed to model additional predictors of cyberbullying victimization drawing on a more diverse range of theories. For comparison, previous studies that have regressed cyberbullying victimization on attachment anxiety together with other predictor variables like self-esteem and depression have derived an *R*^2^ of 0.05 (Canestrari et al., 2021) and 0.14 (Varghese and Pistole, 2017) respectively. Our R^2^ value of 0.09 is in between and therefore sensible. Another possible reason for the less than satisfactory variance explained could be the diverse designs of visibility of different SNS platforms in China unaccounted for in the current study. For instance, posts on WeChat moments can only been seen by one’s WeChat friends whereas posts on Weibo are made visible to the public by default, which lead to different affordances for social media self-disclosure and cyberbullying. Future cyberbullying research involving Chinese respondents should have separate question sections for SNS platforms with different visibility designs.

Limitations aside, this study advances our understanding about the etiology of cyberbullying victimization in the Chinese context and has several implications for the identification of victims and planning targeted protective intervention.

First, as social media self-disclosure constitutes the main risk factor and mediating mechanism for females’ cyberbullying victimization, preventive programs should target females with high attachment anxiety with a focus on educating them on the risks of indiscreet social media self-disclosure and advising them on privacy settings and personal boundaries in online interactions. Indeed, this approach might suffice to minimize females’ risk of cyberbullying victimization resulting from attachment anxiety, because after accounting for social media self-disclosure, attachment anxiety no longer predicts females’ cyberbullying victimization, at least in our sample.

In contrast, for males, the effect of attachment anxiety on cyberbullying victimization is not mediated by social media self-disclosure at all, which means the abovementioned educational approach focused on social media self-disclosure is unlikely to be effective for intervening with males’ cyberbullying victimization. Therefore, further research is warranted to identify the mechanism mediating the effect of attachment anxiety on cyberbullying victimization in Chinese males. While that line of research is underway, action can be taken by leveraging the broader social and cultural context of China to intervene with cyberbullying. For instance, the collectivist culture places a strong emphasis on social cohesion and interdependence, which may influence how individuals navigate social interactions online. On one hand, people from collectivist culture may attach greater importance to generating social gratifications on SNSs than those from individualist culture (Trepte et al., 2017). This may lead individuals to seek validation and approval within their online communities, potentially creating an environment where cyberbullying behaviors can occur. But on the other hand, the sense of social responsibility ingrained in collectivist cultures may also serve as a protective factor against cyberbullying victimization (Zhan et al., 2022). Therefore, interventions for cyberbullying among Chinese college students may focus on further nurturing and reinforcing this sense of social responsibility by encouraging individuals to stand up for victims and provide assistance within their capabilities when witnessing cyberbullying incidents. Additionally, educational initiatives can be designed to emphasize the collective wellbeing and mutual respect that underpin these values and the pivotal role of an inclusive and secure online environment in affording individual social gratifications.

## 6. Conclusion

In summary, our study unveils a moderated mediation model that sheds light on the intricate dynamics of cyberbullying victimization. The findings underscore the mediating role of social media self-disclosure and the moderating role of gender in the relationship between attachment anxiety and cyberbullying. In light of these insights, interventions should be culturally sensitive, recognizing the distinct sociocultural norms and expectations that may influence self-disclosure behaviors and cyberbullying dynamics in China. By tailoring interventions to the distinct characteristics of both genders, we can better equip individuals to navigate the digital landscape while fostering a safer online environment for all.

## Data availability statement

The original contributions presented in the study are included in the article/supplementary material, further inquiries can be directed to the corresponding author.

## Ethics statement

The studies involving human participants were reviewed and approved by the Institutional Review Board of Shanghai Jiao Tong University. Written informed consent to participate in this study was provided by the participants.

## Author contributions

XY: Data curation, Formal analysis, Methodology, Software, Writing – original draft, Writing – review & editing. YH: Formal analysis, Funding acquisition, Writing – original draft, Writing – review & editing. BL: Funding acquisition, Supervision, Writing – review & editing.
